# Risk prediction models for postoperative infections in patients with hip fractures: a systematic review and critical appraisal

**DOI:** 10.3389/fmed.2026.1849317

**Published:** 2026-07-27

**Authors:** Jiye Pan, Juan Shi, Yating Ai, Ming Lu, Huahui Wang

**Affiliations:** 1 School of Nursing, Hubei University of Chinese Medicine, Wuhan, China; 2 Wuhan Hospital of Traditional Chinese Medicine, Wuhan, China; 3 Affiliated Guoyi Hospital of Hubei University of Chinese Medicine, Wuhan, China

**Keywords:** hip fracture, postoperative infection, prediction model, predictor, systematic review

## Abstract

**Background:**

To systematically evaluate the quality and performance of predictive models for postoperative infection risk following hip fractures, to identify reliable tools for clinical practice and provide an evidence-based foundation for the development of higher-quality predictive models in the future.

**Methods:**

A systematic search was conducted on nine databases to retrieve relevant publications, from their inception up to 1 February 2026. Two researchers independently screened the literature and extracted data. They assessed the model bias and applicability using the Predictive Model Risk of Bias Assessment Tool (PROBAST) and the Checklist for Reporting on Multivariate Predictive Models for Individual Prognosis or Diagnosis-Artificial Intelligence (TRIPOD+AI).

**Results:**

A total of 17 articles were included, covering 21 predictive models, with postoperative infection rates ranging from 1.61 to 24.56%. A meta-analysis of 11 high-frequency predictive factors revealed that hypoproteinemia, diabetes, pulmonary disease, ASA classification, smoking, indwelling catheter duration, age, and surgical duration were independent risk factors, while gender and albumin were not statistically significant. Furthermore, the area under the curve (AUC) for the included models ranged from 0.699 to 0.946. While most models performed well, all 17 studies were rated as having a high risk of bias by PROBAST, and the reporting quality of all studies according to TRIPOD+AI was relatively low, primarily due to retrospective study designs, regional bias, inadequate data analysis, insufficient external validation, and a lack of transparency in the research process.

**Conclusion:**

Current predictive models generally demonstrate good overall predictive performance; however, most models suffer from issues such as single-center development, insufficient external validation, and methodological limitations. In the future, more multicenter, large-sample prospective studies should be conducted, and strategies for variable handling and model validation should be optimized to improve the generalizability and clinical translation of predictive models.

**Systematic review registration:**

https://www.crd.york.ac.uk/PROSPERO/view/CRD420261289616, identifier (CRD420261289616).

## 1 Introduction

Hip fractures are commonly referred to as “the last fracture of one’s life” and affect predominantly the elderly. As the population ages, the incidence of hip fractures is rising year after year (1). It is anticipated that by 2050, the number of hip fractures globally will increase dramatically from 1.31 million in 1990 to 6.26 million (2–4). Surgery is the preferred treatment for hip fractures; however, surgery is frequently linked with a variety of comorbidities that can have a major impact on patient outcomes. Postoperative infections are among the most prevalent consequences, consisting mostly of surgical site infections, pulmonary infections, and urinary tract infections (5). Concomitant postoperative infections can significantly hinder patient recovery, lengthen hospital stays, raise treatment expenses, and even result in patient death (6). Studies have indicated that surgical site infections, pulmonary infections, and urinary tract infections increase the mortality rate within 90 days of primary hip replacement surgery by 2.4-fold, 10.7-fold, and 1.6-fold, respectively (7, 8). As a result, building a predictive model for the likelihood of postoperative infection can help identify high-risk individuals early on, allowing for the proactive deployment of essential treatments to minimize postoperative infections and enhance patient outcomes.

Postoperative infections following hip fracture surgery are associated with a variety of factors. Researchers who developed a predictive model for the risk of postoperative infection after a first hip fracture found that cardiovascular and cerebrovascular diseases, liver disease, hematocrit, duration of surgery, the ratio of platelet count to mean platelet volume, and high-sensitivity C-reactive protein are independent risk factors (9). Another study, however, suggests that age, gender, and comorbidities (such as respiratory diseases, hypertension, diabetes, and cardiovascular and cerebrovascular diseases) are associated with postoperative infections following hip fractures (10). Due to the diverse number of predictive factors included in different models and the varying variable selection strategies, such as single-factor analysis, multi-factor analysis, and LASSO, it is difficult for clinical practice to establish a unified screening indicator (11). Meanwhile, the construction methods of the models also vary. Traditional logistic regression algorithms are simple to compute and highly interpretable, making them suitable for analyzing small datasets, while emerging machine learning algorithms offer higher accuracy and stronger generalization capabilities when handling complex, nonlinear data. Different algorithms have their own characteristics, and their predictive performance also varies (12). Therefore, this study will undertake a comprehensive evaluation of risk prediction models for postoperative infections following hip fractures based on the PROBAST framework. The goal is to synthesize and analyze high-frequency predictors, provide recommendations for identifying and screening possible predictors, and provide a solid foundation for selecting the best risk prediction model.

## 2 Methods

### 2.1 Study registration

This study strictly adheres to the Preferred Items for Systematic Reviews and Meta-Analyses (PRISMA) guidelines (13), and has already been registered on the PROSPERO website (ID: CRD420261289616). The registration date is January 12, 2026.

### 2.2 Search strategy

We systematically and comprehensively searched 9 databases, including: PubMed, Embase, Web of Science Core Collection, CINAHL, Cochrane Library, China National Knowledge Infrastructure (CNKI), Wanfang Database, China Biomedical Literature Database (CBM) and VIP Database, from their inception up to 1 February 2026, aiming to identify studies on the development or validation of prediction models for the risk of postoperative infection in hip fractures. Concurrently, supplementary searches were conducted on the list of references included in the study to ensure comprehensiveness. We used the MeSH (Medical Subject Headings) terms and free text terms in several combinations to increase sensitivity. The MeSH terms used in PubMed were “hip fracture,” “intertrochanteric fracture,” “subtrochanteric fracture,” “trochanteric fracture,” “femur trochlear fracture,” “infections,” “prediction model,” “risk prediction model,” “risk factors,” “predictive value of tests,” “risk assessment,” “appraisal,” “logistic model,” “nomogram.” Comprehensive details regarding the search strategy can be found in Supplementary Table S2. Additionally, based on the CHARMS checklist (14), the key elements of this system evaluation were defined using the PICOTS framework (Population, Intervention, Comparator, Outcome, Time, Setting). This framework shaped the clarification of review objectives, establishes the search strategy and defines the inclusion/exclusion criteria. A thorough delineation of the PICOTS framework is presented in Table 1.

**Table 1:** PICOTS framework for study selection.

PICOTS	Explanation
Population	Patients with hip fracture
Intervention	Studies involving the development or validation of multivariable risk prediction models for postoperative infections (predictors≥2)
Comparator	Standard clinical assessment
Outcome	Postoperative infections
Timing	Unrestricted prediction horizon
Setting	The anticipated use of the risk prediction model is to individualize the prediction of postoperative infections in patients with hip fractures, promoting the implementation of preventive measures to improve survival outcomes

### 2.3 Inclusion and exclusion criteria

Include studies that meet all the following criteria:(1) Population: Patients with hip fracture;(2) Study design: Observational design;(3) Prediction model type: development or validation of postoperative infections prediction models;(4) Publication: Chinese or English-language publications.

The studies were excluded if any of the following criteria: (1) Studies involving only independent risk factors did not establish a comprehensive prediction model;(2) Reviews, commentaries, conference abstracts, case reports, or duplicate publications;(3) Predictors of the model<2;(4) Despite efforts to contact the authors via email and other means, full-text documents could not be obtained, or the information provided was incomplete.

### 2.4 Study selection and screening

The literature screening process was conducted independently by two reviewers (J. Y. P. and J. S.), evaluating the relevance of literature metadata using a PICOTS framework. To ensure the rigor and applicability of the research methodology, full-text reviews were conducted on studies preliminarily meeting the inclusion criteria. Any disagreements arising during the screening or extraction process were first discussed and resolved by two researchers. If consensus could not be reached after discussion, a third researcher (Y. T. A.) made the final decision. All disagreements and the process by which they were resolved were documented for review. Interrater agreement was quantified using the unweighted Cohen’s kappa (*κ*) statistic. While adhering to the transparent reporting of a Multivariate Predictive Models for Individual Prognosis or Diagnosis-Artificial Intelligence (TRIPOD+AI) statement (15), giving priority to conducting research that clearly develops, validates or optimizes a multivariate prediction model capable of stratifying individual postoperative infection risks in patients with hip fractures. The inter-rater reliability was good, with a concordance Kappa value of 0.85 (95% CI: 0.80–0.90).

### 2.5 Data extraction

Developed a standardized Excel spreadsheet for data extraction and aggregation based on the Critical Appraisal and Data Extraction for Systematic Reviews of Prediction Modelling Studies (CHARMS) checklist (14) derived from predictive modeling research. The data extraction process strictly complies with CHARMS principles, extracting content principally includes: Authors, publication year, country, Study design, Study population, Data source, event rates, Candidate variables, missing data handling methods, sample size, Model development methods, Calibration methods, Validation methods, Model performance and presentation, etc. To ensure data accuracy, two researchers (J. Y. P. and J. S.) independently extracted data, then cross-validated their findings. Unresolved discrepancies were discussed and analyzed, with a third researcher (M. L.) making the final determination.

### 2.6 Risk of bias and applicability assessment

Two reviewers (J. Y. P. and J. S.) evaluated the risk of bias and applicability of included studies using the Prediction Model Risk of Bias Assessment Tool (PROBAST) (16). The risk of bias assessment primarily covered four key domains: study population, predictors, outcomes, and statistical analysis. Applicability primarily included: study population, predictors, and outcomes. Questions were answered using the response options “yes/probably yes, no/probably no, no information.” This method assists researchers in systematically identifying potential bias risks and quantitatively evaluating them. The specific assessment criteria are as follows: if all responses are “yes/probably yes” the risk of bias is considered low; if at least one response is “no/probably no” the risk of bias is considered high; if “no information” appears in the responses but other questions are answered as “yes/probably yes” the risk of bias status is unclear. Following the initial reviews, cross-validation is conducted. Where discrepancies persist after discussion and analysis, the matter is referred to by a third researcher (Y. T. A.) for adjudication.

### 2.7 Data synthesis

We conducted a meta-analysis using Stata 18.0 software to assess the predictive performance and associated predictive factors of the included studies. The effect size for predictive performance was expressed as the area under the curve (AUC) for each model and its 95% confidence interval (CI), while the effect size for predictive factors was expressed as the odds ratio (OR) and its 95% confidence interval (CI). A *p*-value of < 0.05 was considered statistically significant. The *I*^2^ statistic was used to assess heterogeneity among studies. When heterogeneity was negligible (*p* > 0.05, *I*^2^ < 50%), a fixed-effects model was used; if heterogeneity remained significant (*p* < 0.05, *I*^2^ ≥ 50%), a random-effects model was used for the meta-analysis.

## 3 Results

### 3.1 Search results

**Figure 1:**
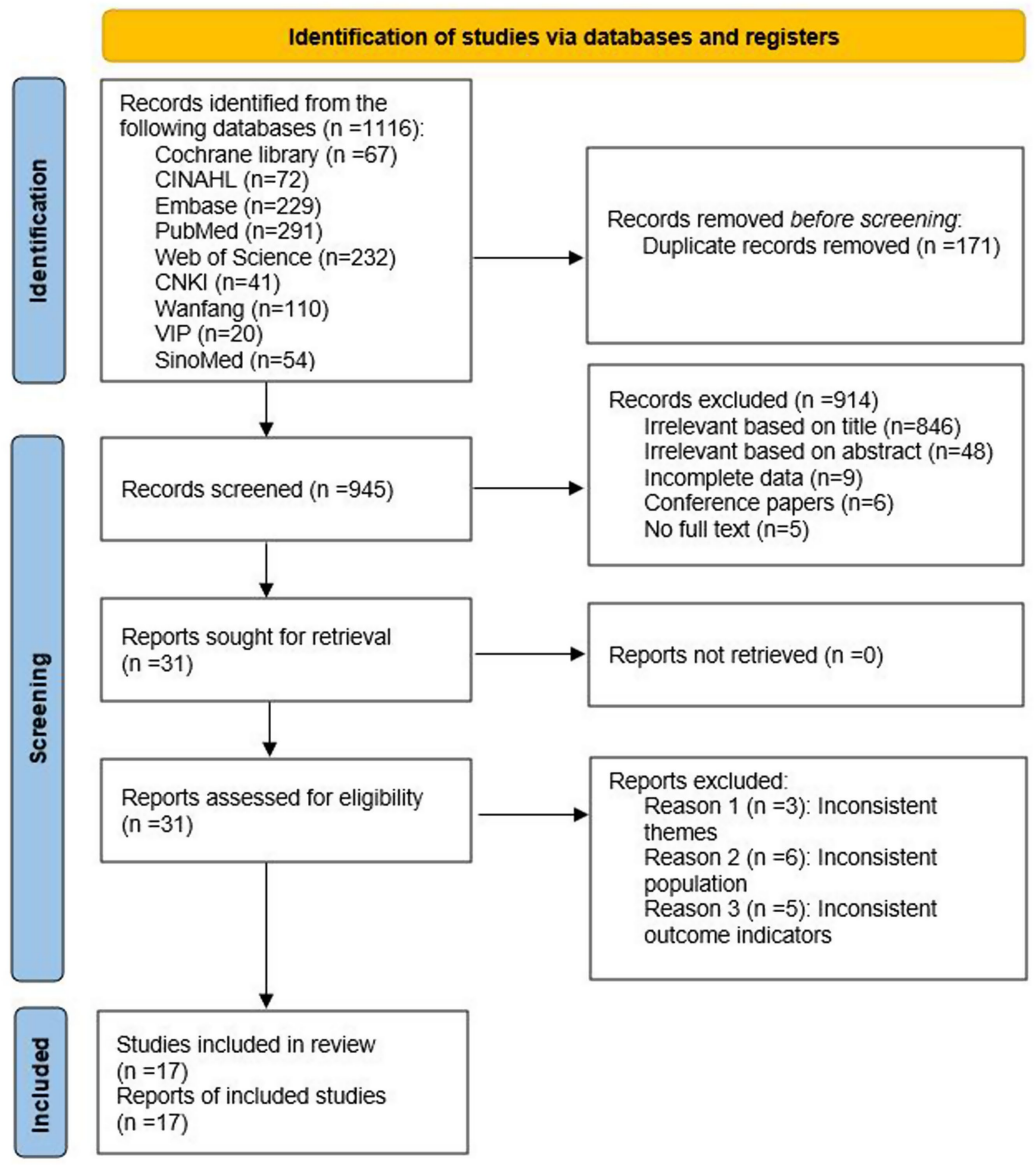
Preferred reporting items for systematic reviews and meta-analyses (PRISMA) flowchart of literature search and selection.

Figure 1 is the Preferred Reporting Items for Systematic Reviews and Meta-Analyses (PRISMA) flowchart, which details the search process, exclusion criteria, and results. Based on the established search strategy, we systematically identified 1,116 articles potentially relevant to predictive models for postoperative infections following hip fractures. After excluding 171 duplicate articles, 945 articles underwent title and abstract screening. Following this screening process, a total of 31 articles were selected for further full-text review, and ultimately, 17 eligible studies were included.

### 3.2 Study characteristics

Table 2 provides a detailed description of the baseline characteristics of the included studies. All 17 studies were conducted in China (2022–2025), seven of them were published in English (9, 17–22), this cohort comprises 16 retrospective cohort studies and 1 case–control study (23), this includes 16 single-center studies and 1 multicenter study (18), the study population included patients with hip fractures (*n* = 13), specifically those with femoral neck fractures or intertrochanteric fractures (*n* = 4); in these studies, the incidence of postoperative hip fracture infections ranged from 1.61 to 24.56%.

**Table 2:** Overview and characteristics of included studies (*n* = 17).

First author (Year)	Study design	Study population	Data source	Events cases/Full sample	Main outcome
Shi et al. (31)(2024)	Retrospective cohort study	Patients with femoral neck fracture or intertrochanteric fractures undergoing surgical treatment ≥80 years	A Grade A Tertiary Hospital in Weifang	12.12%	Healthcare-associated infection
Wang et al. (23)(2024)	Retrospective case control	Patients with hip fractures undergoing surgical treatment ≥60 years	Wuxi Traditional Chinese Medicine Hospital	9.18%	Pulmonary infection
Li et al. (25)(2025)	Retrospective cohort study	Patients with hip fractures undergoing surgical treatment age 60–89	Henan Provincial Second People’s Hospital	23.75%	Pulmonary infection
Zhang et al. (26)(2022)	Retrospective cohort study	Patients with hip fractures undergoing surgical treatment ≥60 years	Shenzhen Second People’s Hospital	5.89%	Pulmonary infection
Wang et al. (24)(2022)	Retrospective cohort study	Patients with hip fractures undergoing surgical treatment ≥60 years	Beijing Shijitan Hospital	23.84%	Urinary tract infection
Wang et al. (32)(2024)	Retrospective cohort study	Patients with hip fractures undergoing surgical treatment ≥65 years	Beijing Luhe Hospital	10.97%	Urinary tract infection
Yu et al. (27)(2023)	Retrospective cohort study	Patients with hip fractures undergoing surgical treatment ≥65 years	A Grade A Tertiary Hospital in Tianjin	17.68%	Urinary tract infection
Yang et al. (28)(2024)	Retrospective cohort study	Patients with hip fractures undergoing surgical treatment ≥60 years	Zhengzhou Orthopedic Hospital	10.94%	Pulmonary infection
Zhao et al. (29)(2024)	Retrospective cohort study	Patients with intertrochanteric fractures undergoing surgical treatment ≥60 years	Tangshan Second Hospital	8.22%	Surgical site infection
Mu et al. (30)(2024)	Retrospective cohort study	Patients with hip fractures undergoing surgical treatment ≥18 years	Peking Union Medical College Hospital	1.61%	Surgical site infection
Guo et al. (20)(2025)	Retrospective cohort study	Patients with hip fractures undergoing surgical treatment >60 years	The Third Hospital of Hebei Medical University	3.22%	Surgical site infection
Cheng et al. (9) (2022)	Retrospective cohort study	Patients with acute hip fractures undergoing primary total hip arthroplasty or hemiarthroplasty >65 years	The Third Hospital of Hebei Medical University	3.87%	Postoperative infection
Tang et al. (21)(2023)	Retrospective cohort study	Patients with hip fractures undergoing surgical treatment ≥60 years	Dandong Central Hospital	24.56%	Urinary tract infection
Huang et al. (17) (2023)	Retrospective cohort study	Patients with unilateral femoral neck fracture or intertrochanteric fractures undergoing surgical treatment ≥65 years	Shanghai Tenth Peoples’ Hospital	8.63%	Pulmonary infection
Peng et al. (22) (2023)	Retrospective cohort study	Patients with hip fractures undergoing surgical treatment ≥65 years	West China Hospital of Sichuan University	20.09%	Postoperative infection
Dai et al. (18) (2024)	Retrospective cohort study	Patients with hip fractures undergoing surgical treatment ≥65 years	Nanjing First Hospital and the Fourth Affiliated Hospital of Nanjing Medical University	13.86%	Pulmonary infection
Xi et al. (19) (2025)	Retrospective cohort study	Patients with intertrochanteric fracture of the femur undergoing surgical treatment	Southern Central Hospital of Yunnan Province	6.38%	Postoperative infection

### 3.3 Establishment and performance of risk prediction models

The 17 included studies comprised 21 models, one of which was a study on model development (24), 10 Studies on Model Development and Internal Validation (9, 19, 21, 22, 25–30), include 1,000-fold bootstrap, 10-fold cross-validation, and a combination of the two; four of these are related to model development and external research (17, 23, 31, 32), two projects focusing on model development and internal and external validation (18, 20). Regarding the choice of modeling methods, while one study (18) selected logistic regression (LR), random forest (RF), categorical boosting (Catboost), extreme gradient boosting (XGB), and light gradient boosting machine (LGBM), the remaining 16 studies all employed logistic regression for modeling. The range of potential predictor variables is 5 to 39, and variable selection methods include LASSO, univariate analysis, multivariate analysis, forward stepwise regression, and logistic regression. Regarding the handling of missing data, two studies used mean imputation (17, 27), one study employed multiple interpolation methods (22), one study used the K-Nearest Neighbor (KNN) algorithm (18), two studies directly excluded incomplete case studies (26, 31), the remaining 11 studies did not report the status of missing data or how they were handled (9, 19–21, 23–25, 28–30, 32). In terms of data presentation, 14 studies used line charts (9, 17, 19–23, 26–32), two studies used mathematical formulas to express (24, 25), one study employed web risk calculator (18). The results are shown in Table 3.

**Table 3:** The establishment of a predictive model for postoperative infection risk in hip fractures.

First author(year)	Sample size (MG/IV/EV)	Candidate variables		Missing data		Modelling method	Selection of candidate predictors	Model types
		No.	Continuous variables processing method	No.	Methods of handling			
Shi et al. (31)(2024)	439/−/188	26	Some converted to categorical variables	10%	Direct Delete	LR	SFA + MFA	D + V
Wang et al. (23)(2024)	305/−/133	5	Maintain continuity	-	-	LR	SFA + MFA	D + V
Li et al. (25)(2025)	80/−/−	8	Maintain continuity	-	-	LR	LASSO	D + V
Zhang et al. (26)(2022)	939/402/−	18	Maintain continuity	20%	Direct Delete	LR	LASSO	D + V
Wang et al. (24)(2022)	151/−/−	12	All converted to categorical variables	-	-	LR	SFA	D + V
Wang et al. (32)(2024)	1012/−/101	6	All converted to categorical variables	-	-	LR	SFA + MFA	D + V
Yu et al. (27)(2023)	673/−/−	15	Some converted to categorical variables	-	Mean imputation	LR	FSA	D + V
Yang et al. (28)(2024)	417/104/−	9	Maintain continuity	-	-	LR	SFA + MFA	D + V
Zhao et al. (29)(2024)	284/142/−	5	Maintain continuity	-	-	LR	FSR	D + V
Mu et al. (30)(2024)	783/335/−	11	Maintain continuity	-	-	LR	LASSO	D + V
Guo et al. (20)(2025)	1430/−/307	11	Some converted to categorical variables	-	-	LR	LR	D + V
Cheng et al. (9) (2022)	1,084/−/−	11	Some converted to categorical variables	-	-	LR	SFA + MFA	D + V
Tang et al. (21)(2023)	632/268/−	39	Some converted to categorical variables	-	-	LR	SFA + MFA	D + V
Huang et al. (17) (2023)	1008/−/305&195	18	All converted to categorical variables	-	Mean imputation	LR	SFA + MFA	D + V
Peng et al. (22) (2023)	476/201/−	35	Maintain continuity	25%	Multiple imputation	LR	SFA + MFA	D + V
Dai et al. (18) (2024)	373/125/124	24	Maintain continuity	10%	KNN	LR; RF; Catboost; XGB; LGBM	LASSO	D + V
Xi et al. (19) (2025)	395/169/−	16	All converted to categorical variables	–	–	LR	SFA + MFA	D + V

–, unreported; SFA, single-factor analysis; MFA, multi-factor analysis; FSR, forward stepwise regression; D + V, development + validation; No., number; LR, logistic regression; KNN, K-Nearest Neighbor; LASSO, the least absolute shrinkage and selection operator; RF, random forest; XGB, extreme gradient boosting; LGBM, light gradient boosting machine; Catboost, Categorical boosting; MG, modelling group; IV, internal validation; EV, External validation.

In terms of model performance, discriminatory power was reported using the area under the receiver operating characteristic curve (AUC), which ranged from 0.699 to 0.946, indicating good model performance. Regarding calibration methods, 9 studies used the Hosmer-Lemeshow test (9, 17, 19, 20, 23, 24, 27, 29, 31), six studies used calibration curves (18, 21, 22, 25, 28, 30), two studies did not mention the calibration method (26, 32). In terms of overall performance, only 2 studies reported Brier scores (18, 20), which ranged from 0.030 to 0.112, indicating that the overall probabilistic prediction error of the models is at a good to acceptable level. In terms of clinical utility, 8 studies only completed the graphical plotting of decision curve analysis (9, 19–21, 23, 26, 27, 30), but none of them reported specific net benefit values in numerical form. In terms of classification efficacy, 11 studies reported sensitivity (range: 0.605–0.924) and specificity (range: 0.235–0.9219) (9, 17–20, 24–27, 29, 32), but almost all results were obtained based on the optimal cutoff values of their respective internal validation sets. There is considerable variation in threshold selection, making direct cross-study comparison difficult. Overall, current evidence indicates that prediction models in this field have good discriminative performance, but there are significant deficiencies in calibration reporting and clinical utility assessment. Future research should strengthen the standardized reporting of full-dimensional model performance.

### 3.4 Meta-analysis of logistic regression models and high-frequency predictors

#### 3.4.1 Meta-analysis of logistic regression models

**Figure 2:**
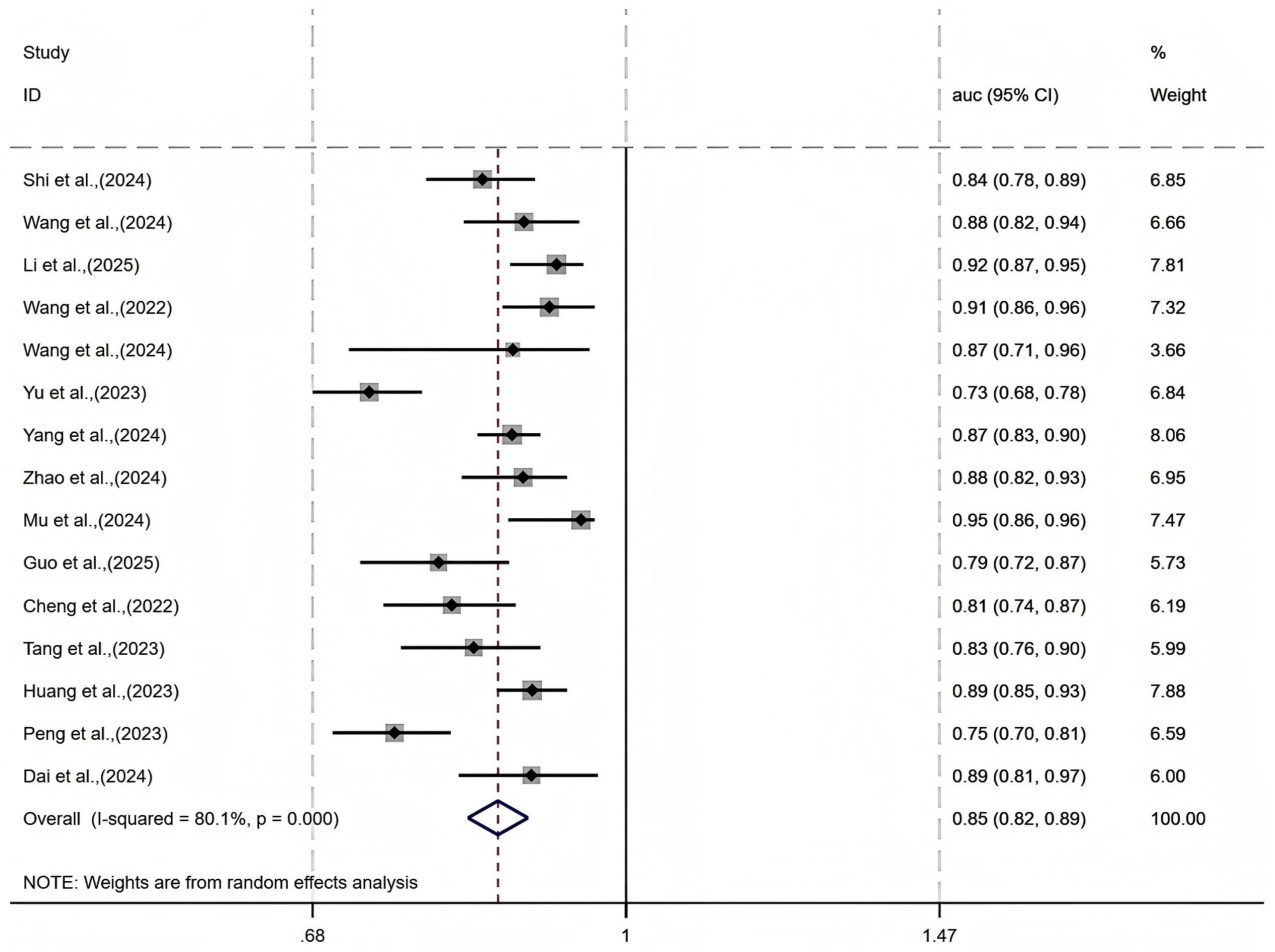
Forest plot of meta-analysis of pooled AUC estimates for 15 LR models.

**Figure 3:**
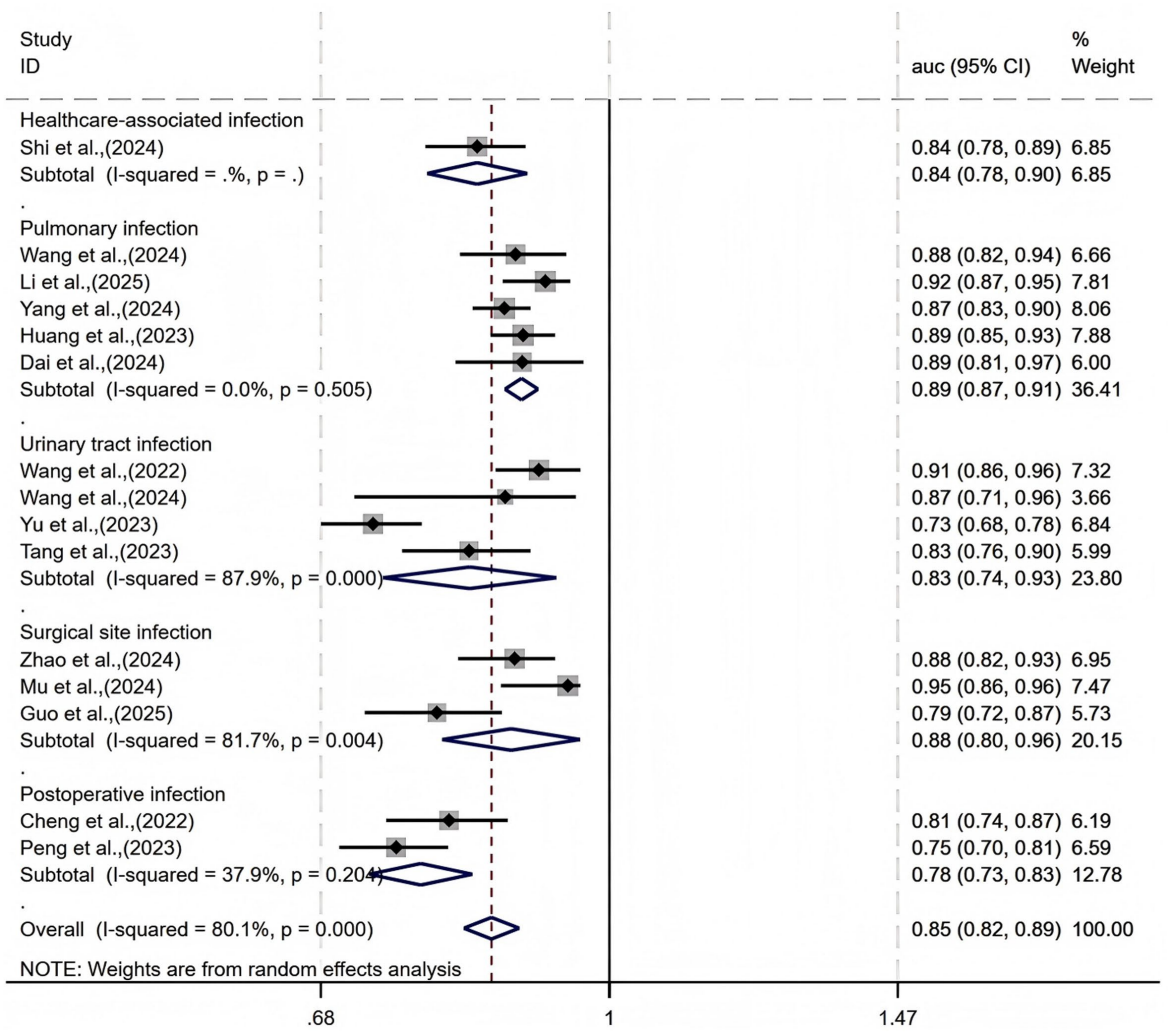
Subgroup analysis of a forest plot of AUC estimates from 15 combined LR models.

We conducted a meta-analysis of models from the modeling group that applied logistic regression algorithms, including 15 models that reported the AUC and its 95% confidence interval (95% CI). Two studies by Zhang et al. (26) and Xi et al. (19) were excluded because they did not report 95% confidence intervals for the AUC values and thus did not meet the criteria for data pooling based on AUC values. The meta-analysis of the 15 studies showed that we pooled all AUCs using a random-effects model, yielding a pooled AUC of 0.85 (95% CI: 0.82, 0.89) and an *I*^2^ of 80.1% (*p* < 0.001) (Figure 2), indicating high heterogeneity. Furthermore, we conducted a subgroup analysis of the logistic regression models by site of infection. The results showed that the effect sizes in the Pulmonary infection subgroup (0.89; 95% CI: 0.87, 0.91) and the Surgical site infection subgroup (0.88; 95% CI: 0.80, 0.96) were more significant than those in the Healthcare-associated infection subgroup (0.84; 95% CI: 0.78, 0.90) and the urinary tract infection subgroup (0.78; 95% CI: 0.73, 0.83) (Figure 3). These results suggest that there are significant differences in model effect sizes across different infection sites, indicating that the site of infection may be a potential effect modifier on the diagnostic performance of the model. Nevertheless, interpretation of these subgroup differences requires considerable caution. First, the limited number of included studies within each infection subtype may yield unstable estimates due to insufficient statistical power. Second, the diagnostic criteria for the same infection type are not fully consistent across included studies, and minor discrepancies between some studies may introduce cross-study classification bias. Third, and most critically, the distribution of included studies across study designs and center types is uneven within each subgroup. Therefore, future research should unify infection diagnostic criteria across studies, adopt multi-center prospective designs to validate these subgroup differences, and quantify the magnitude of the influence of study design characteristics on AUC estimates via meta-regression analysis. Sensitivity analysis was conducted using a stepwise elimination approach. The results showed that the pooled effect size did not change substantially, indicating that the conclusions of this study are robust (see Supplementary Figure S1).

#### 3.4.2 Meta-analysis of high-frequency predictors

As shown in Table 4, the models included in the study comprised 4 to 9 predictors, totaling 45 independent predictors. High-frequency predictors (appearing in ≥4 models) included: age (12 models), gender (6 models), ASA classification (5 models), diabetes (5 models), operative time (4 models), albumin (4 models), indwelling catheter duration (4 models), hypoproteinemia (4 models), smoking (4 models), chronic lung disease (4 models), and C-reactive protein (4 models). A meta-analysis was conducted on high-frequency predictors, and Table 5 (Supplementary Figure S2) presents the results of the meta-analysis regarding the association between 11 high-frequency predictors and the outcome. In the included analyses, 10 predictors demonstrated statistical significance (*p* < 0.05). Among these, hypoproteinemia (OR = 5.59, 95% CI: 2.68–11.66), pulmonary disease (OR = 5.42, 95% CI: 1.38–21.24), and diabetes (OR = 3.05, 95% CI: 1.42–6.56) were the strongest predictors associated with the outcome. Additionally, ASA classification (OR = 2.59, 95% CI: 1.26–5.30), smoking (OR = 2.81, 95% CI: 1.98–3.98), and duration of indwelling urinary catheterization (OR = 2.35, 95% CI: 1.11–4.97) also showed significant associations of moderate strength. Although the effect sizes for age (OR = 1.31, 95% CI: 1.18–1.45) and operative time (OR = 1.13, 95% CI: 1.02–1.26) were small, they were also statistically significant. The effect size for C-reactive protein was close to 1 but did not reach statistical significance (OR = 1.01, 95% CI: 1.00–1.03, *p* = 0.058). Notably, the pooled results for the predictor “smoking” showed no heterogeneity (*I*^2^ = 0, *p* = 0.552), indicating high consistency among studies; therefore, a fixed-effects model was used. The remaining predictors all exhibited significant heterogeneity (*I*^2^ ranging from 69.2 to 95.4%), so a random-effects model was used for pooling. Gender (OR = 1.49, 95% CI: 0.56–3.95, *p* = 0.423) and albumin levels (OR = 0.89, 95% CI: 0.75–1.06, *p* = 0.183) were not statistically significantly associated with postoperative infections.

**Table 4:** Performance and predictive factor analysis of a postoperative infection risk prediction model for hip fractures.

First author(year)	Model performance							Validation methodology		number of predictors	Predictors	Model presentation
	AUC/C-index	Sensitivity	Specificity	Accuracy	Brier score	Calibration method	Clinical benefit					
Shi et al. (31)(2024)	0.838^a^ 0.805^c^	–	–	–	–	H-L test	–	–	EV	6	Admission to the ICU; age; albumin level; gender; ASA classification; perioperative impairment of consciousness	Nomogram
Wang et al. (23)(2024)	0.882^a^ 0.851^b^	–	–	–	–	H-L test + CC	DCA	–	EV	5	preoperative length of hospital stay; time spent in bed; white blood cell count; high-sensitivity C-reactive protein; serum sodium level	Nomogram
Li et al. (25)(2025)	0.918^a^ 0.937^b^	0.9031	0.9219	–	–	CC	–	1000Bootstrap	–	5	age; preoperative procalcitonin levels; concomitant diabetes; duration of surgery; invasive procedures	Mathematical Formula
Zhang et al. (26)(2022)	0.699^a^ 0.781^b^	0.924	0.571	0.905	-	-	DCA	IV	-	5	age; gender; white blood cell count; Serum Albumin /Globulin ratio; Hemoglobin	Nomogram
Wang et al. (24)(2022)	0.910	0.806	0.843	–	–	H-L test	–	–	–	6	gender; age; anemia; hypoproteinemia; indwelling urinary catheter for >3 days; time from admission to surgery >48 h	Mathematical Formula
Wang et al. (32)(2024)	0.87	0.8378	0.9077	–	–	–	–	–	EV	4	hypoproteinemia; electrolyte imbalance; pneumonia; diabetes	Nomogram
Yu et al. (27)(2023)	0.729^a^ 0.718^b^	0.605	0.235	-	-	H-L test + CC	DCA	IV	-	5	diabetes; age; electrolyte imbalance; gender; indwelling urinary catheter > 3 days	Nomogram
Yang et al. (28)(2024)	0.869^a^ 0.884^b^	–	–	–	–	CC	–	IV	–	5	age; red blood cell distribution width; albumin; CRP; COPD	Nomogram
Zhao et al. (29)(2024)	0.881^a^ 0.878^b^	0.8333	0.8385	-	-	H-L test	-	IV	-	5	age; diabetes, hypoproteinemia; BMI; failure to follow medical instructions regarding hand hygiene	Nomogram
Mu et al. (30)(2024)	0.946	–	–	–	–	CC	DCA	IV	–	4	gender; smoking status; BMI; preoperative albumin levels	Nomogram
Guo et al. (20)(2025)	0.749^a^ 0.774^b^ 0.804^c^	0.839	0.638	–	0.030	H-L test + CC	DCA	1000Bootstrap	EV	6	age; ASA classification; surgical delay≥6 days; surgical duration>180 min; PAR ≥ 6.6; SII ≥ 541.1	Nomogram
Cheng et al. (9) (2022)	0.807^a^ 0.784^b^	0.738	0.743	-	-	H-L test + CC	DCA	1000Bootstrap	-	7	heart disease; cerebrovascular disease; liver disease; time to surgery; hematocrit; PMR; HCRP	Nomogram
Tang et al. (21)(2023)	0.829^a^ 0.803^b^	–	–	–	–	H-L test + CC	DCA	IV	–	9	age; female gender; diabetes; fracture type; surgery type; bedridden time; blood glucose >6.10 mmol/L; albumin <35 g/L; indwelling urinary catheter duration	Nomogram
Huang et al. (17) (2023)	0.891^a^ 0.881^c^ 0.843^c^	0.828	0.786	–	–	H-L test	–	–	EV	9	age; time from fracture to surgery; history of smoking; ASA classification; COPD; hypoproteinemia; RDW; mechanical ventilation time; check-in ICU	Nomogram
Peng et al. (22) (2023)	0.752^a^ 0.723^b^	–	–	–	–	CC	–	IV	–	8	albumin; cholesterol; blood phosphorus; high-density lipoprotein; surgery type; smoking; ASA classification; chronic pulmonary disease	Nomogram
Dai et al. (18) (2024)	Catboost0.895^a^ 0.835^b^ 0.894^c^	0.830^a^ 0.786^b^ 0.765^c^	0.825^a^ 0.816^b^ 0.854^c^	0.826^a^ 0.784^b^ 0.844^c^	0.067^a^ 0.112^b^ 0.070^c^	CC	–	10-fold cross-validation	EV	7	age; CRP; preoperative length of stay; mFI-5; smoking; preoperative SpO2; ASA physical status	web risk calculator
Xi et al. (19) (2025)	0.827^a^ 0.810^b^	0.792	0.803	-	-	H-L test + CC	DCA	1000Bootstrap + 10-fold cross-validation	-	5	age; ICU admission; urinary catheter time>24 h; surgery time > 2 h; use of steroids	Nomogram

–, unreported; CC, calibration curve; H-L test, Hosmer-Lemeshow Goodness of fit test; BMI, body mass index; ASA, American Society of Anesthesiologists; PAR, platelet count/ albumin; SII, platelet count×neutrophil count/lymphocyte count; HCRP, high-sensitivity C-reactive protein; PMR, the platelet-to-mean platelet volume ratio; RDW, red cell distribution width; CRP, C-reactive protein; mFI-5, modified five-item frailty index; Preoperative SpO2, Preoperative oxygen saturation; DCA, Decision curve analysis.

^a^Modelling group.

*^b^* Internal validation group.

^c^External validation group.

**Table 5:** Outcomes of the meta-analysis of high-frequency predictors.

predictors	Number of studies	Overall effect			Heterogeneity		Analysis model
		OR (95% CI)	*Z*	*p*	*I*^2^ (%)	*p*	
Age	12	1.31(1.18, 1.45)	5.14	< 0.001	95.4	< 0.001	Random-effects
ASA classification	5	2.59(1.26, 5.30)	2.60	0.009	70.2	0.009	Random-effects
Gender	5	1.49(0.56, 3.95)	0.80	0.423	89.5	< 0.001	Random-effects
Diabetes	5	3.05(1.42, 6.56)	2.85	0.004	94.9	< 0.001	Random-effects
Duration of surgery	4	1.13(1.02, 1.26)	2.26	0.024	69.2	0.021	Random-effects
C-reactive protein	4	1.01(1.00, 1.03)	1.89	0.058	82.6	0.001	Random-effects
Smoking	4	2.81(1.98, 3.98)	5.81	< 0.001	0	0.552	Fixed-effects
Albumin	4	0.89 (0.75, 1.06)	1.33	0.183	76.0	0.006	Random-effects
Duration of indwelling urinary catheterization	4	2.35(1.11, 4.97)	2.23	0.026	87.1	< 0.001	Random-effects
Hypoproteinemia	4	5.59(2.68, 11.66)	4.59	< 0.001	79.2	0.002	Random-effects
Lung diseases	4	5.42(1.38, 21.24)	2.42	0.015	88.4	< 0.001	Random-effects

### 3.5 Risk of Bias and applicability

**Figure 4:**
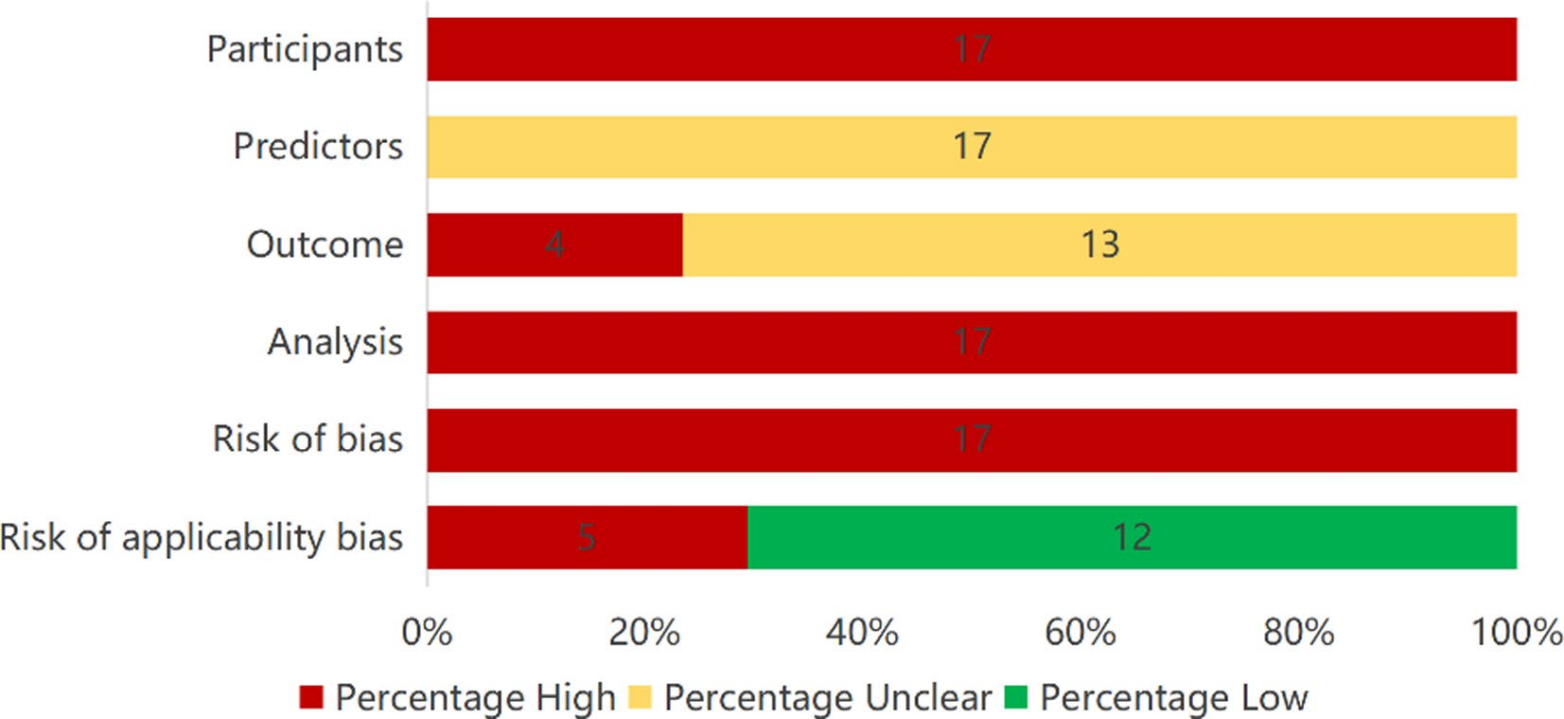
PROBAST risk of bias assessment: Distribution of risk of bias results according to PROBAST criteria.

An assessment of the risk of bias in the 17 included studies revealed that, overall, all studies were at high risk of bias. Study design: all 17 studies were at high risk of bias, primarily due to the study types being retrospective cohort studies or case–control studies, for prognostic models, we generally recommend prospective longitudinal cohort studies, as they minimize selection bias and measurement bias (33). Predictor assessment: the risk of bias for all 17 studies was unclear, mainly because it was not reported whether predictors were assessed under blinded conditions; Outcome assessment: four studies were at high risk of bias (19, 22, 28, 32), the primary reason is that infections are diagnosed based solely on non-standardized clinical symptoms (such as cough, fever, and frequent urination) rather than international standards or laboratory test results, which can easily lead to misclassification; 13 studies had an unclear risk of bias (9, 17, 18, 20, 21, 23–27, 29–31), the main issue is the lack of information regarding the time intervals between the assessment of predictive factors and the determination of outcomes; Analysis: all 17 studies were at high risk of bias, primarily because most studies did not explicitly report their methods for handling missing data or the adequacy of their sample sizes. Results are shown in Supplementary Table S3 and Figure 4.

Results of the applicability assessment: Four studies were identified as having a high risk of applicability (17, 19, 29, 31), 13 studies were classified as having a low risk of applicability (9, 18, 20–28, 30, 32). The four studies included only patients with femoral neck fractures or intertrochanteric fractures (17, 19, 29, 31), and Shi et al. (31) restricted the age to ≥80 years. Results are shown in Supplementary Table S3 and Figure 4.

### 3.6 Transparent reporting

**Figure 5:**
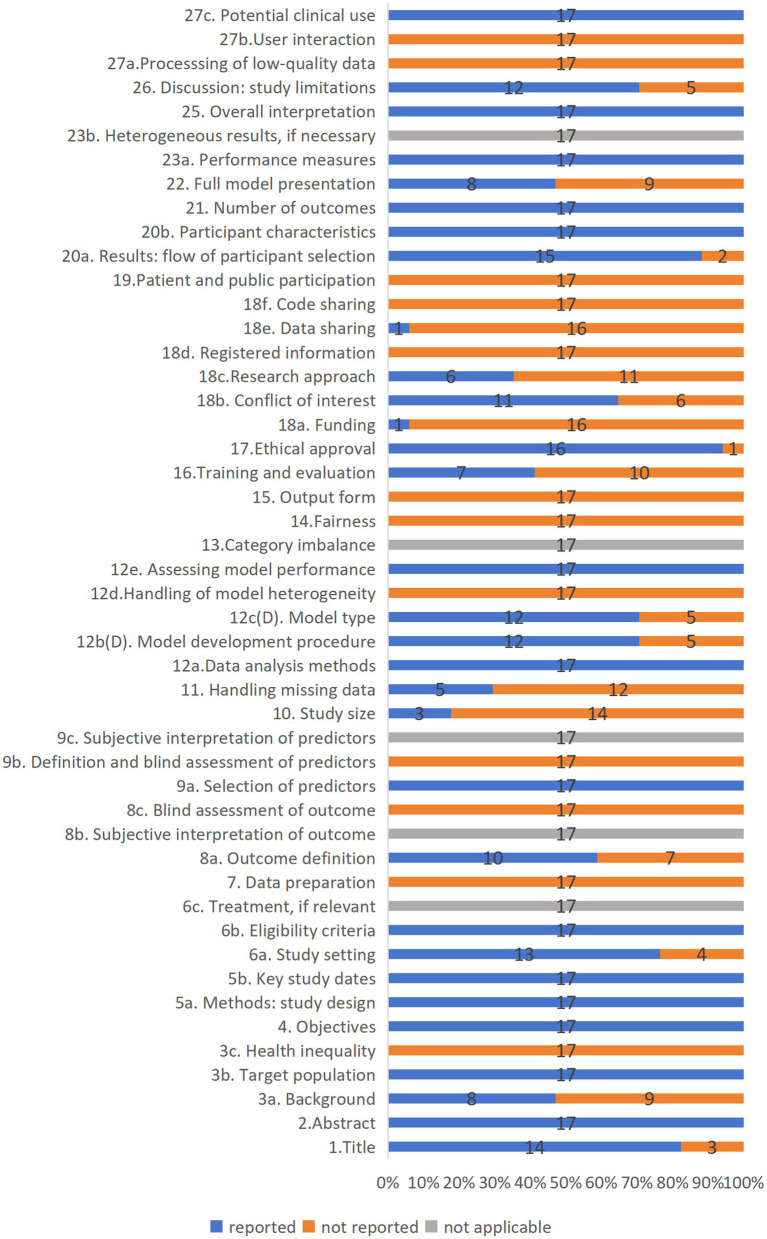
Assessment of included studies according to the TRIPOD+AI (Transparent Reporting of a multivariable prediction model for Individual Prognosis or Diagnosis) statement.

The results of the report quality assessment are shown in Supplementary Table S4 and Figure 5; overall, the 17 included studies had low report quality. Titles and Abstracts (items 1–2): three studies did not specify in their titles whether the study was for the development or validation of a predictive model (22, 26, 29); Introduction (items 3–4): nine studies did not adequately explain the underlying principles of predictive model development or validation (17, 20, 22–25, 28, 30, 31), all 17 studies clearly described the target population and intended use of the predictive model, but did not report on health inequalities; Methods (items 5–17): four studies did not mention the study setting (19, 20, 25, 32), none of the 17 studies provided specific details regarding outcome measures, blinded assessment of predictive factors, or risk stratification; only 3 studies explained the rationale for their sample sizes (26, 27, 31), 6 studies explain how to handle missing data (17, 18, 22, 26, 27, 31), five studies did not report their internal model validation methods (17, 23, 24, 31, 32), one study did not provide information regarding ethical review (27), none of the 17 studies mentioned the methods used to address model fairness; Open Science (Item 18): only 6 studies provided Supplementary information or online calculators (9, 17, 18, 20, 22, 26), one study details the sources of research funding and the role of funders in the research (20), six studies did not disclose all authors’ conflicts of interest (19, 24, 25, 27, 29, 32), none of the 17 studies provided detailed information on study registration, data availability, or the availability of analysis code; Patient and Public Involvement (Item 19): none of the 17 studies mentioned whether patients or the public were involved in the study process; Results (Items 20–24): most studies adequately described the characteristics of the study participants and the model-building process, but only 8 studies met the requirements for detailed model reporting (17, 18, 21, 24–27, 31), the remaining nine studies did not describe how the predictive models were used (9, 19, 20, 22, 23, 28–30, 32); Discussion (Items 25–27): With the exception of five studies that did not discuss their limitations (23, 24, 29, 31, 32), the remaining studies have effectively addressed their limitations and outlined their potential for future clinical applications.

## 4 Discussion

This study employed the CHARMS framework to systematically identify and evaluate predictive models for postoperative infection risk in patients with hip fractures, conducting a comprehensive search of major international and Chinese databases for studies that developed and validated such models. A total of 17 studies were ultimately included; however, only 6 of these studies included external validation of the models. Since 64.71% (11/17) of the studies did not perform external validation, this may result in insufficient generalizability of the models, increased risk of overfitting, and reduced reliability and applicability of the models. To address the prevalent issue of the lack of external validation, this problem can be resolved from multiple dimensions and perspectives. Specifically, methods including the adoption of a standardized multi-center data sharing mechanism, a privacy-preserving federated learning framework, temporal validation and geographical validation applicable to single-center cohorts, and model updating strategies can be employed to examine the stability and generalizability of models, thereby enhancing the clinical translational value of the models (34). The models were primarily presented using line charts (9, 17, 19–23, 26–32), it also includes mathematical formulas and online risk calculators (18, 24, 25). Of the 21 models, 17 reported the area under the receiver operating characteristic curve (AUC), ranging from 0.699 to 0.946. Among these, 94.12% (*n* = 16) of the models had an AUC > 0.7, indicating good predictive performance. A total of 16 studies used logistic regression (LR) for modeling, while Dai et al. (18) employed LR, RFC, Catboost, XGBoost, and LGBM, and utilized Shapley Additive Explanations (SHAP) to visualize and rank the importance of potential risk factors. They also developed a web-based risk calculator that can identify high-risk patients requiring early intervention upon data input, providing direction and insights for the clinical application of big data modeling. As seen in the preceding analysis, logistic regression is a commonly used model-building method, primarily employed for classification problems; the models it produces are simple and highly interpretable. In contrast, XGBoost is often used to optimize generalization performance when dealing with high-dimensional data and class imbalance. Golinelli et al. (35) used machine learning to predict surgical site infections following orthopedic surgery; the results showed that XGBoost outperformed other algorithms, with an AUC of 0.768. Biavardi et al. (36) developed a predictive model for prosthetic joint infection following hip replacement for femoral fractures. They trained and compared six algorithms (logistic regression, random forest, support vector machine, multilayer perceptron, XGBoost, and stacking ensemble). All models had AUC > 0.84, with XGBoost and RF demonstrating the best discriminatory ability, with AUCs of 0.889 and 0.881, respectively, significantly outperforming traditional logistic regression. In summary, different algorithms each have their own advantages, and the application of machine learning offers greater potential for model development. When conducting future clinical studies, the optimal algorithm for building predictive models should be selected after comprehensively considering the research type and objective.

Due to differences in study design, the predictors ultimately included in each model varied. Among the 21 models, the number of potential predictors ranged from 5 to 39, while the number of predictors ultimately included ranged from 4 to 9. The four most frequently identified predictors were age, sex, ASA classification, and diabetes. This study found that hypoproteinemia, diabetes, pulmonary disease, ASA classification, smoking, indwelling catheter duration, age, and surgical duration are independent risk factors for postoperative infection following hip fracture surgery (*p* < 0.05). Additionally, previous research suggests that age, ASA classification, and diabetes are independent risk factors for postoperative infection following hip fracture surgery (37, 38), which is consistent with the findings of our study. Elderly patients with hip fractures experience functional decline and weakened immune systems, which significantly reduces their ability to defend against and eliminate pathogens. Additionally, their postoperative recovery is slower, their metabolic function declines, and they spend more time bedridden, all of which contribute to an increased risk of postoperative infection (39). Patients with a history of lung disease typically exhibit characteristics such as tracheal stenosis and increased airway secretions, postoperatively, bacteria in the upper airway and oropharynx can transform into pathogenic bacteria and enter the lower airway, concurrently, patients experience reduced organ function and weakened defense mechanisms, and are prone to developing hypoproteinemia, which leads to tissue edema and pulmonary edema, this, in turn, reduces the levels of immunoglobulins, adversely affecting the body’s immune response, tissue repair, and wound healing, thereby increasing the likelihood of postoperative pulmonary infections following hip fracture surgery (40–42). Patients with hip fractures complicated by diabetes exhibit elevated plasma osmolarity and impaired phagocytic function in white blood cells, which prevents them from effectively eliminating bacteria. Concurrent tissue metabolic disorders further impair wound healing, thereby increasing the risk of infection (43). According to studies, patients classified as ASA Class III-IV are more likely to experience postoperative complications, with a pulmonary infection rate 3.99 times higher than that of patients classified as ASA Class III or lower (38). Long-term smokers experience increased respiratory secretions, which raises the risk of lung infections, it also impairs microvascular circulation, immune function, tissue perfusion, and oxygenation, thereby slowing wound healing and increasing the risk of infection (44, 45). It is well known that prolonged indwelling urinary catheters promote bacterial colonization of the urinary tract. Studies have shown that, over time, fibrinogen and other host factors deposit on the catheter surface, providing a substrate for microbial adhesion and biofilm formation; therefore, the longer the catheter remains in place, the greater the risk of infection (46). Longer surgical procedures expose the patient’s skin to the environment for a longer period of time, increasing the risk of pathogens entering the surgical incision and the risk of trauma and stress to the patient, thereby raising the risk of infection (47).

The above discussion provides a biological explanation for the clinical significance of each predictive factor. However, it should be cautiously noted that some of the aforementioned predictive factors (particularly age and diabetes) exhibited extreme heterogeneity in this meta-analysis. This stems from significant differences among the original studies in how they defined the same predictive factor, selected cutoff values, and defined the types of infection outcomes they focused on, which directly affected the precision and generalizability of the pooled effect estimates. Therefore, the current pooled results should be regarded as an exploratory summary of trend effects rather than definitive conclusions. Clinical interpretation must be made with caution, taking into account the definitional differences across the original studies. This finding also points to an important direction for future research in this field: it is recommended that future studies on predictive models adhere to the TRIPOD + AI statement, clearly defining the methodology and rationale for cutoff values when reporting predictive factors, in order to enhance the comparability of results across studies and the reliability of meta-analyses.

The PROBAST assessment indicates a high risk of bias (particularly in the analysis domain), but this is precisely where the value of systematic reviews lies—in revealing the weaknesses in the existing evidence base and pointing the way toward high-quality future research. Regarding predictors, the risk of bias was unclear in all studies, primarily because the articles did not specify whether the predictors were assessed under blinded conditions. This raises the possibility that the assessment process was influenced by outcome information; if assessors were aware of the outcome status in advance, they might unconsciously favor predictors associated with the outcome, thereby overestimating the importance of certain predictors, leading to model overfitting and reducing the model’s predictive accuracy (16). In terms of results, four studies were assessed as having a high risk of bias because the outcome measures were based solely on unstandardized clinical symptoms (19, 22, 28, 32), for all other factors, the risk of bias is unclear; the procedures for assessing outcome measures should be standardized, including consistent definitions, measurement tools, scoring systems, and cutoff values; otherwise, this will hinder the model’s implementation and application in clinical practice. In terms of analysis, all 17 studies were at high risk of bias, primarily because most studies did not explicitly report their methods for handling missing data or the adequacy of their sample sizes. The handling of missing data is critical to model performance and generalizability. Common methods include direct deletion, multiple imputation, and machine learning-based approaches; however, directly excluding patients with missing data should generally be avoided, as this may lead to selection bias and reduced statistical power. Sample size is closely related to the accuracy and stability of the model; insufficient sample size increases the risk of model overfitting (48). In addition to the issues related to missing data and sample size mentioned above, we also identified several other methodological flaws in the included studies, which collectively led to a high risk of bias in the analysis. First, some studies exhibited eternal time bias, meaning that predictors were assessed after admission, while infection outcomes occurred during hospitalization, and the time window for assessing predictors was not clearly defined; Second, in studies where the outcome assessors were not blinded to predictor information, confirmation bias may have been present; third, in some studies, predictor selection and model building were based on the same dataset without the use of an independent validation set, resulting in circular reasoning, which could lead to overly optimistic estimates of model performance. In terms of adaptability assessment, four studies were deemed to pose a high risk of applicability due to differences in the inclusion and exclusion criteria for study participants (17, 19, 29, 31), this is primarily due to limitations such as fracture type and age; future studies could conduct subgroup analyses to reduce heterogeneity among the various models.

The results of the quality assessments for all studies were generally poor, primarily due to a failure to report blinding procedures, describe methods for handling missing data, mention sample size calculation formulas, or give sufficient consideration to equity. None of the 17 studies reported specific details regarding the blinding of predictors and outcome measures, which undermined the reliability of the models; only 6 studies described how missing data were handled (17, 18, 22, 26, 27, 31), included direct delete, mean imputation, multiple imputation, KNN, researchers should report instances of missing data in detail; simply deleting missing data may result in a large number of outliers in the included data, thereby affecting the accuracy and reliability of the results (49). Research indicates that machine learning can make the most of complex, high-dimensional data, thereby enhancing statistical performance (50). Only three studies reported their sample size calculation methods (26, 27, 31), researchers should clearly define the methods used to determine sample size during the model development and validation phases, demonstrate that the sample size is sufficient to address the research questions, and ensure the statistical power of the model. Common sample size calculation methods include the traditional 10 EPV approach and Riley’s four-step method (51). All 17 included studies did not mention any methods for model fairness calibration, which represents a major gap that urgently needs to be addressed in the current field of clinical prediction modeling, as fairness runs through all critical links including data collection, method selection and performance evaluation. By analyzing health inequalities across different populations, predictive performance across different subgroups, and feedback from beneficiary groups such as patients, the public, and healthcare professionals, researchers can effectively enhance the equity of model design and improve the model’s accuracy and generalizability (15). Meanwhile, the present study finds that the vast majority of included studies did not report calibration or decision curve analysis, which reflects the prevalent deficiency in the completeness of model evaluation for predictive model research in this field. We recommend that future studies should strictly comply with the TRIPOD+AI statement. When reporting the AUC, it is mandatory to supplement calibration metrics such as calibration plots or calibration slopes, as well as decision curve analysis with explicit reporting of the net benefit value, to ensure a complete evidence chain for the model from “statistically valid” to “clinically useful.”

## 5 Strengths and limitations

A strength of this study is its strict adherence to the PRISMA and CHARMS frameworks for standardized reporting, with two researchers independently conducting literature screening, PROBAST assessments, and transparency reporting to ensure the objectivity and accuracy of the evaluation results. However, this study has several potential limitations. First, by limiting the scope to literature published in Chinese or English, high-quality studies in other languages may have been overlooked. Second, given differences among countries and regions in perioperative infection control strategies, antibiotic usage guidelines, and patient baseline characteristics, the effects of the risk factors identified in this study (such as hypoproteinemia and ASA classification) may vary across different populations. Finally, although most studies conducted internal validation, only a few chose to perform external validation using independent cohorts; the model’s generalizability and stability have not yet been fully tested.

## 6 Conclusion

Currently, while risk prediction models for postoperative infections following hip fractures generally demonstrate good overall predictive performance and high discriminatory power, they are also associated with high risks of overall model bias and applicability bias, as well as issues such as low-quality reporting, methodological flaws, and insufficient external validation. These factors severely limit the clinical translation value of these models. It is worth noting that hypoproteinemia, diabetes, pulmonary disease, ASA classification, smoking, indwelling catheter duration, age, and surgical duration are independent risk factors for postoperative hip fracture infections and should be prioritized in future model development. It is recommended that future researchers familiarize themselves with the PROBAST + AI checklist and adhere to the reporting guidelines outlined in the TRIPOD + AI statement to reduce the risk of bias and improve report quality. By conducting multicenter, large-sample prospective studies and external validation studies, researchers should develop predictive models with good stability, high reliability, and applicability to a broader population, ultimately achieving the precise identification and early intervention of high-risk patients for postoperative hip fracture infections.

## Data Availability

The original contributions presented in the study are included in the article/Supplementary material, further inquiries can be directed to the corresponding author.
