# Supplementary material for: Risk prediction models for postoperative infections in patients with hip fractures: a systematic review and critical appraisal
Source: Front Med (Lausanne). 2026 Jul 27;13:1849317. doi: 10.3389/fmed.2026.1849317 (PMC13453693; doi:10.3389/fmed.2026.1849317)
Supplement: Supplementary file 1 [file Table_1.docx]

**Supplementary Materials**

Table S1. PRISMA Checklist

| **Section and topic** | **Item #** | **Checklist item** | **Location where item is reported** |
| --- | --- | --- | --- |
| **Title** | | |  |
| Title | 1 | Identify the report as a systematic review. | **Manuscript,** Page 1 |
| **Abstract** | | |  |
| Abstract | 2 | See the PRISMA 2020 for Abstracts checklist. | **Manuscript,** Page 1 |
| **Introduction** | | |  |
| Rationale | 3 | Describe the rationale for the review in the context of existing knowledge. | **Manuscript,** Introduction |
| Objectives | 4 | Provide an explicit statement of the objective(s) or question(s) the review addresses. | **Manuscript,** Introduction |
| **Methods** | | |  |
| Eligibility criteria | 5 | Specify the inclusion and exclusion criteria for the review and how studies were grouped for the syntheses. | **Manuscript,** Methods: Inclusion and Exclusion Criteria |
| Information sources | 6 | Specify all databases, registers, websites, organisations, reference lists and other sources searched or consulted to identify studies. Specify the date when each source was last searched or consulted. | **Manuscript,** Methods: Search Strategy |
| Search strategy | 7 | Present the full search strategies for all databases, registers and websites, including any filters and limits used. | **Manuscript,** Methods: Search Strategy  **Supplementary** Table S2 |
| Selection process | 8 | Specify the methods used to decide whether a study met the inclusion criteria of the review, including how many reviewers screened each record and each report retrieved, whether they worked independently, and if applicable, details of automation tools used in the process. | **Manuscript,** Methods: Study selection and screening |
| Data collection process | 9 | Specify the methods used to collect data from reports, including how many reviewers collected data from each report, whether they worked independently, any processes for obtaining or confirming data from study investigators, and if applicable, details of automation tools used in the process. | **Manuscript,** Methods: Study selection and screening |
| Data items | 10a | List and define all outcomes for which data were sought. Specify whether all results that were compatible with each outcome domain in each study were sought (e.g. for all measures, time points, analyses), and if not, the methods used to decide which results to collect. | **Manuscript,** Methods: Data extraction |
|  | 10b | List and define all other variables for which data were sought (e.g. participant and intervention characteristics, funding sources). Describe any assumptions made about any missing or unclear information. | **Manuscript,** Methods: Data extraction |
| Study risk of bias assessment | 11 | Specify the methods used to assess risk of bias in the included studies, including details of the tool(s) used, how many reviewers assessed each study and whether they worked independently, and if applicable, details of automation tools used in the process. | **Manuscript,** Risk of Bias and Applicability Assessment |
| Effect measures | 12 | Specify for each outcome the effect measure(s) (e.g., risk ratio, mean difference) used in the synthesis or presentation of results. | **Manuscript,** Methods: Data synthesis |
| Synthesis methods | 13a | Describe the processes used to decide which studies were eligible for each synthesis (e.g., tabulating the study intervention characteristics and comparing against the planned groups for each synthesis (item #5)). | **Manuscript,** Methods: Data synthesis |
|  | 13b | Describe any methods required to prepare the data for presentation or synthesis, such as handling of missing summary statistics, or data conversions. | **Manuscript,** Methods: Data synthesis |
|  | 13c | Describe any methods used to tabulate or visually display results of individual studies and syntheses. | **Manuscript,** Methods: Data synthesis |
|  | 13d | Describe any methods used to synthesise results and provide a rationale for the choice(s). If meta-analysis was performed, describe the model(s), method(s) to identify the presence and extent of statistical heterogeneity, and software package(s) used. | **Manuscript,** Methods: Data synthesis |
|  | 13e | Describe any methods used to explore possible causes of heterogeneity among study results (e.g., subgroup analysis, metaregression). | **Manuscript,** Methods: Data synthesis |
|  | 13f | Describe any sensitivity analyses conducted to assess robustness of the synthesised results. | **Manuscript,** Methods: Data synthesis |
| Reporting bias assessment | 14 | Describe any methods used to assess risk of bias due to missing results in a synthesis (arising from reporting biases). | Not applicable |
| Certainty assessment | 15 | Describe any methods used to assess certainty (or confidence) in the body of evidence for an outcome. | Not applicable |
| **Results** | | |  |
| Study selection | 16a | Describe the results of the search and selection process, from the number of records identified in the search to the number of studies included in the review, ideally using a flow diagram | **Manuscript,**  Results: Figure 1 |
|  | 16b | Cite studies that might appear to meet the inclusion criteria, but which were excluded, and explain why they were excluded. | Not applicable |
| Study characteristics | 17 | Cite each included study and present its characteristics. | **Manuscript,**  Study characteristics  , Table 2 |
| Risk of bias in studies | 18 | Present assessments of risk of bias for each included study. | **Manuscript, Supplementary** Table S3 |
| Results of individual studies | 19 | For all outcomes, present, for each study: (a) summary statistics for each group (where appropriate) and (b) an effect estimate and its precision (e.g., confidence/credible interval), ideally using structured tables or plots. | **Manuscript,** results: Table2-3 |
| Results of syntheses | 20a | For each synthesis, briefly summarise the characteristics and risk of bias among contributing studies. | **Manuscript,** Transparent Reporting |
|  | 20b | Present results of all statistical syntheses conducted. If meta-analysis was done, present for each the summary estimate and its precision (e.g., confidence/credible interval) and measures of statistical heterogeneity. If comparing groups, describe the direction of the effect. | **Manuscript,** Meta-Analysis of Logistic Regression Models and High-Frequency Predictors |
|  | 20c | Present results of all investigations of possible causes of heterogeneity among study results. | **Manuscript,** Meta-Analysis of Logistic Regression Models and High-Frequency Predictors |
|  | 20d | Present results of all sensitivity analyses conducted to assess the robustness of the synthesised results. | **Manuscript,** Meta-Analysis of Logistic Regression Models and High-Frequency Predictors |
| Reporting biases | 21 | Present assessments of risk of bias due to missing results (arising from reporting biases) for each synthesis assessed. | Not applicable |
| Certainty of evidence | 22 | Present assessments of certainty (or confidence) in the body of evidence for each outcome assessed. | Not applicable |
| **Discussion** | | |  |
| Discussion | 23a | Provide a general interpretation of the results in the context of other evidence. | **Manuscript,** Discussion |
|  | 23b | Discuss any limitations of the evidence included in the review. | **Manuscript,** Strengths and Limitations |
|  | 23c | Discuss any limitations of the review processes used. | **Manuscript,** Strengths and Limitations |
|  | 23d | Discuss implications of the results for practice, policy, and future research. | **Manuscript,** Conclusion |
| **Other information** | | |  |
| Registration and protocol | 24a | Provide registration information for the review, including register name and registration number, or state that the review was not registered. | **Manuscript,** Methods: Study registration |
|  | 24b | Indicate where the review protocol can be accessed, or state that a protocol was not prepared. | **Manuscript,** Methods |
|  | 24c | Describe and explain any amendments to information provided at registration or in the protocol. | Not applicable |
| Support | 25 | Describe sources of financial or nonfinancial support for the review, and the role of the funders or sponsors in the review. | **Manuscript,** At the very end of the text |
| Competing interests | 26 | Declare any competing interests of review authors. | **Manuscript,** At the very end of the text |
| Availability of data, code, and other materials | 27 | Report which of the following are publicly available and where they can be found; template data collection forms; data extracted from included studies; data used for all analyses; analytic code; any other materials used in the review. | Not applicable |

Table S2. Search strategy conducted on 02/01/2026

| Database | Result | Date of search | Search string |
| --- | --- | --- | --- |
| Cochrane library | 67 | 02/01/2026 | #1 (hip fracture):ti,ab,kw OR (intertrochanteric fracture):ti,ab,kw OR (subtrochanteric fracture):ti,ab,kw OR (trochanteric fracture):ti,ab,kw OR (femur trochlear fracture):ti,ab,kw (Word variations have been searched) 8875  #2 (femoral trochlear fracture):ti,ab,kw OR (femoral neck fracture):ti,ab,kw OR (femur neck fracture):ti,ab,kw (Word variations have been searched) 3455  #3 MeSH descriptor: [Hip Fractures] explode all trees 2633  #4 #1 OR #2 OR #3 9894  #5 MeSH descriptor: [Infections] explode all trees 111668  #6 (infections):ti,ab,kw (Word variations have been searched) 83044  #7 #5 OR #6 143119  #8 (prediction model*):ti,ab,kw (Word variations have been searched) 8866  #9 (risk prediction model*):ti,ab,kw (Word variations have been searched) 4310  #10 (risk factors):ti,ab,kw (Word variations have been searched) 105222  #11 (predictive value of tests):ti,ab,kw (Word variations have been searched) 11280  #12 (risk prediction*):ti,ab,kw (Word variations have been searched) 7748  #13 (risk assessment*):ti,ab,kw (Word variations have been searched) 94109  #14 (appraisal*):ti,ab,kw (Word variations have been searched) 3034  #15 (risk assessor*):ti,ab,kw (Word variations have been searched) 2383  #16 (risk calculation*):ti,ab,kw (Word variations have been searched) 4041  #17 (predictive model*):ti,ab,kw (Word variations have been searched) 12242  #18 (logistic model*):ti,ab,kw (Word variations have been searched) 20020  #19 (nomogram*):ti,ab,kw (Word variations have been searched) 2096  #20 #8 OR #9 OR #10 OR #11 OR #12 OR #13 OR #14 OR #15 OR #16 OR #17 OR #18 OR #19 203809  #21 #4 AND #7 AND #20 |
| CINAHL | 72 | 02/01/2026 | #1 MH hip fractures 13437  #2 SU (Intertrochanteric Fracture* OR Subtrochanteric Fracture* OR Trochanteric Fracture* OR Femur Trochlear Fracture* OR Femoral Trochlear Fracture* OR Femoral Neck Fracture* OR Femur Neck Fracture*) 433  #3 #1 OR #2 13807  #4 SU (infection) 280265  #5 XB (prediction model* OR risk prediction model* OR risk factors OR predictive value of tests OR risk prediction* OR risk assessment* OR appraisal* OR risk assessor* OR risk calculation* OR predictive model* OR logistic model* OR nomogram*) 375206  #6 #3 AND #4 AND #5 |
| Embase | 229 | 02/01/2026 | #1 'hip fracture':ab,ti OR 'intertrochanteric fracture':ab,ti OR 'subtrochanteric fracture':ab,ti OR 'trochanteric fracture':ab,ti OR 'femur trochlear fracture':ab,ti OR 'femoral trochlear fracture':ab,ti OR 'femur neck fracture':ab,ti 30391  #2 'infection':ab,ti 2052381  #3 'prediction model*':ab,ti OR 'risk prediction model*':ab,ti OR 'risk factors':ab,ti OR 'predictive value of tests':ab,ti OR 'risk prediction*':ab,ti OR 'risk assessment*':ab,ti OR 'appraisal*':ab,ti OR 'risk assessor*':ab,ti OR 'risk calculation*':ab,ti OR 'predictive model*':ab,ti OR 'logistic model*':ab,ti OR 'nomogram*':ab,ti 1404461  #4 #1 AND #2 AND #3 |
| PubMed | 291 | 02/01/2026 | #1 hip fractures [MeSH Terms] 32093  #2 Hip Fractures [Title/Abstract] OR Intertrochanteric Fracture*[Title/Abstract] OR Subtrochanteric Fracture*[Title/Abstract] OR Trochanteric Fracture*[Title/Abstract] OR Femur Trochlear Fracture*[Title/Abstract] OR Femoral Trochlear Fracture*[Title/Abstract] OR Femoral Neck Fracture*[Title/Abstract] OR Femur Neck Fracture*[Title/Abstract] 24042  #3 #1 OR #2 39867  #4 Infection*[Title/Abstract] 2009205  #5 prediction model*[Title/Abstract] OR risk prediction model*[Title/Abstract] OR risk factors[Title/Abstract] OR predictive value of tests[Title/Abstract] OR risk prediction*[Title/Abstract] OR risk assessment*[Title/Abstract] OR appraisal*[Title/Abstract] OR risk assessor*[Title/Abstract] OR risk calculation*[Title/Abstract] OR predictive model*[Title/Abstract] OR logistic model*[Title/Abstract] OR nomogram*[Title/Abstract]  1014539  #6 #3 AND #4 AND #5 |
| Web of Science | 232 | 02/01/2026 | #1 TS= (Hip Fractures OR Intertrochanteric Fracture* OR Subtrochanteric Fracture* OR Trochanteric Fracture* OR Femur Trochlear Fracture* OR Femoral Trochlear Fracture* OR Femoral Neck Fracture* OR Femur Neck Fracture*) 73161  #2 TS=(Infection*) 2198127  #3 TL= (prediction model* OR risk prediction model* OR risk factors OR predictive value of tests OR risk prediction* OR risk assessment* OR appraisal* OR risk assessor* OR risk calculation* OR predictive model* OR logistic model* OR nomogram*) 533973  #4 #1 AND #2 AND #3 |
| CNKI | 41 | 02/01/2026 | SU=('髋部骨折' + '转子骨折' + '转子间骨折' + '转子下骨折' + '股骨颈骨折') AND SU= ('感染' + '术后感染') AND TKA=('风险因素' + '预测模型' + '风险预测模型' + '风险预测') |
| Wanfang Database | 110 | 02/01/2026 | 主题:(髋部骨折 OR 转子骨折 OR 转子间骨折 OR 转子下骨折 OR 股骨颈骨折) and 题名或关键词:(感染 OR 术后感染) and 主题:(风险因素 OR 预测模型 OR 风险预测模型 OR 风险预测 OR 列线图) |
| VIP Database | 20 | 02/01/2026 | M=(髋部骨折 OR 转子骨折 OR 转子间骨折 OR 转子下骨折 OR 股骨颈骨折) and M=(感染 OR 术后感染) and M=(风险因素 OR 预测模型 OR 风险预测模型 OR 风险预测 OR 列线图) |
| China Biomedical Literature Database (CBM) | 54 | 02/01/2026 | ( "髋部骨折"[常用字段:智能] OR "转子骨折"[常用字段:智能] OR "转子间骨折"[常用字段:智能] OR "转子下骨折"[常用字段:智能] OR "股骨颈骨折"[常用字段:智能]) AND ( "感染"[常用字段:智能] OR "术后感染"[常用字段:智能]) AND ( "风险因素"[常用字段:智能] OR "预测模型"[常用字段:智能] OR "风险预测模型"[常用字段:智能] OR "风险预测"[常用字段:智能]) |

Table S3 Assessment results of bias risk and applicability for prediction model risk of bias assessment tool (PROBAST) included in the study

| Study | **risk of bias** | | | | **Applicability** | | | **Overall** | |
| --- | --- | --- | --- | --- | --- | --- | --- | --- | --- |
|  | **Participants** | **Predictors** | **Outcome** | **Analysis** | **Participants** | **Predictors** | **Outcome** | **risk of bias** | **Applicability** |
| Shi et al. (2024) | H | ? | ? | H | H | L | L | H | H |
| Wang et al. (2024) | H | ? | ? | H | L | L | L | H | L |
| Li et al. (2025) | H | ? | ? | H | L | L | L | H | L |
| Zhang et al. (2022) | H | ? | ? | H | L | L | L | H | L |
| Wang et al. (2022) | H | ? | ? | H | L | L | L | H | L |
| Wang et al. (2024) | H | ? | H | H | L | L | L | H | L |
| Yu et al. (2023) | H | ? | ? | H | L | L | L | H | L |
| Yang et al. (2024) | H | ? | H | H | L | L | L | H | L |
| Zhao et al. (2024) | H | ? | ? | H | H | L | L | H | H |
| Mu et al. (2024) | H | ? | ? | H | L | L | L | H | L |
| Guo et al. (2025) | H | ? | ? | H | L | L | L | H | L |
| Cheng et al. (2022) | H | ? | ? | H | L | L | L | H | L |
| Tang et al. (2023) | H | ? | ? | H | L | L | L | H | L |
| Huang et al. (2023) | H | ? | ? | H | H | L | L | H | H |
| Peng et al. (2023) | H | ? | H | H | L | L | L | H | L |
| Dai et al. (2024) | H | ? | ? | H | L | L | L | H | L |
| Xi et al. (2025) | H | ? | H | H | H | L | L | H | H |

Abbreviations: H, high risk; L, low risk; ?, unclear

Table S4 TRIPOD+AI checklist

| First author (year)  item | Shi et al. (2024) | Wang et al. (2024) | Li et al. (2025) | Zhang et al. (2022) | Wang et al. (2022) | Wang et al. (2024) | Yu et al. (2023) | Yang et al. (2024) | Zhao et al. (2024) | Mu et al. (2024) | Guo et al. (2025) | Cheng et al. (2022) | Tang et al. (2023) | Huang et al. (2023) | Peng et al. (2023) | Dai et al. (2024) | Xi et al. (2025) |
| --- | --- | --- | --- | --- | --- | --- | --- | --- | --- | --- | --- | --- | --- | --- | --- | --- | --- |
| 1.Title | Y | Y | Y | N | Y | Y | Y | Y | N | Y | Y | Y | Y | Y | N | Y | Y |
| 2.Abstract | Y | Y | Y | Y | Y | Y | Y | Y | Y | Y | Y | Y | Y | Y | Y | Y | Y |
| 3a. Background | N | N | N | Y | N | Y | Y | N | Y | N | N | Y | Y | N | N | Y | Y |
| 3b. Target population | Y | Y | Y | Y | Y | Y | Y | Y | Y | Y | Y | Y | Y | Y | Y | Y | Y |
| 3c. Health inequality | N | N | N | N | N | N | N | N | N | N | N | N | N | N | N | N | N |
| 4. Objectives | Y | Y | Y | Y | Y | Y | Y | Y | Y | Y | Y | Y | Y | Y | Y | Y | Y |
| 5a. Methods: study design | Y | Y | Y | Y | Y | Y | Y | Y | Y | Y | Y | Y | Y | Y | Y | Y | Y |
| 5b. Key study dates | Y | Y | Y | Y | Y | Y | Y | Y | Y | Y | Y | Y | Y | Y | Y | Y | Y |
| 6a. Study setting | Y | Y | N | Y | Y | N | Y | Y | Y | Y | N | Y | Y | Y | Y | Y | N |
| 6b. Eligibility criteria | Y | Y | Y | Y | Y | Y | Y | Y | Y | Y | Y | Y | Y | Y | Y | Y | Y |
| 6c. Treatment, if relevant | - | - | - | - | - | - | - | - | - | - | - | - | - | - | - | - | - |
| 7. Data preparation | N | N | N | N | N | N | N | N | N | N | N | N | N | N | N | N | N |
| 8a. Outcome definition | N | Y | N | Y | Y | N | Y | N | Y | Y | Y | Y | Y | N | N | Y | N |
| 8b. Subjective interpretation of outcome | - | - | - | - | - | - | - | - | - | - | - | - | - | - | - | - | - |
| 8c. Blind assessment of outcome | N | N | N | N | N | N | N | N | N | N | N | N | N | N | N | N | N |
| 9a. Selection of predictors | Y | Y | Y | Y | Y | Y | Y | Y | Y | Y | Y | Y | Y | Y | Y | Y | Y |
| 9b. Definition and blind assessment of predictors | N | N | N | N | N | N | N | N | N | N | N | N | N | N | N | N | N |
| 9c. Subjective interpretation of predictors | - | - | - | - | - | - | - | - | - | - | - | - | - | - | - | - | - |
| 10. Study size | Y | N | N | Y | N | N | Y | N | N | N | N | N | N | N | N | N | N |
| 11. Handling missing data | Y | N | N | N | N | N | Y | N | N | N | N | N | N | Y | Y | Y | N |
| 12a.Data analysis methods | Y | Y | Y | Y | Y | Y | Y | Y | Y | Y | Y | Y | Y | Y | Y | Y | Y |
| 12b(D). Model development procedure | N | N | Y | Y | N | N | Y | Y | Y | Y | Y | Y | Y | N | Y | Y | Y |
| 12c(D). Model type | N | N | Y | Y | N | N | Y | Y | Y | Y | Y | Y | Y | N | Y | Y | Y |
| 12d.Handling of model heterogeneity | N | N | N | N | N | N | N | N | N | N | N | N | N | N | N | N | N |
| 12e. Assessing model performance | Y | Y | Y | Y | Y | Y | Y | Y | Y | Y | Y | Y | Y | Y | Y | Y | Y |
| 13.Category imbalance | - | - | - | - | - | - | - | - | - | - | - | - | - | - | - | - | - |
| 14.Fairness | N | N | N | N | N | N | N | N | N | N | N | N | N | N | N | N | N |
| 15. Output form | N | N | N | N | N | N | N | N | N | N | N | N | N | N | N | N | N |
| 16.Training and evaluation | Y | N | N | Y | N | N | N | N | Y | Y | N | N | Y | Y | N | Y | N |
| 17.Ethical approval | Y | Y | Y | Y | Y | Y | N | Y | Y | Y | Y | Y | Y | Y | Y | Y | Y |
| 18a. Funding | N | N | N | N | N | N | N | N | N | N | Y | N | N | N | N | N | N |
| 18b. Conflict of interest | Y | Y | N | Y | N | N | N | Y | N | Y | Y | Y | Y | Y | Y | Y | N |
| 18c.Research approach | N | N | N | Y | N | N | N | N | N | N | Y | Y | N | Y | Y | Y | N |
| 18d. Registered information | N | N | N | N | N | N | N | N | N | N | N | N | N | N | N | N | N |
| 18e. Data sharing | N | N | N | N | N | N | N | N | N | N | N | Y | N | N | N | N | N |
| 18f. Code sharing | N | N | N | N | N | N | N | N | N | N | N | N | N | N | N | N | N |
| 19.Patient and public participation | N | N | N | N | N | N | N | N | N | N | N | N | N | N | N | N | N |
| 20a. Results: flow of participant selection | Y | Y | N | Y | Y | Y | Y | Y | N | Y | Y | Y | Y | Y | Y | Y | Y |
| 20b. Participant characteristics | Y | Y | Y | Y | Y | Y | Y | Y | Y | Y | Y | Y | Y | Y | Y | Y | Y |
| 21. Number of outcomes | Y | Y | Y | Y | Y | Y | Y | Y | Y | Y | Y | Y | Y | Y | Y | Y | Y |
| 22. Full model presentation | Y | N | Y | Y | Y | N | Y | N | N | N | N | N | Y | Y | N | Y | N |
| 23a. Performance measures | Y | Y | Y | Y | Y | Y | Y | Y | Y | Y | Y | Y | Y | Y | Y | Y | Y |
| 23b. Heterogeneous results, if necessary | - | - | - | - | - | - | - | - | - | - | - | - | - | - | - | - | - |
| 25. Overall interpretation | Y | Y | Y | Y | Y | Y | Y | Y | Y | Y | Y | Y | Y | Y | Y | Y | Y |
| 26. Discussion: study limitations | N | N | Y | Y | N | N | Y | Y | N | Y | Y | Y | Y | Y | Y | Y | Y |
| 27a.Processsing of low-quality data | N | N | N | N | N | N | N | N | N | N | N | N | N | N | N | N | N |
| 27b.User interaction | N | N | N | N | N | N | N | N | N | N | N | N | N | N | N | N | N |
| 27c. Potential clinical use | Y | Y | Y | Y | Y | Y | Y | Y | Y | Y | Y | Y | Y | Y | Y | Y | Y |

Abbreviations: Y, yes; N, no; -, not applicable.

 Figure S1: Sensitivity Analysis of the AUC Values for LR Models in the Modelling group

Figure S2 Forest plot of meta-analysis of high-frequency predictors

A1, Age

A2, ASA classification

A3, gender

A4, diabetes

A5, surgical duration

A6, C reactive protein

A7, smoking

A8, albumin

A9, urinary catheter time

A10, hypoproteinemia

A11, Pulmonary disease
